# Preemptive Analgesic and Antioxidative Effect of Curcumin for Experimental Migraine

**DOI:** 10.1155/2017/4754701

**Published:** 2017-10-24

**Authors:** Adriana E. Bulboacă, Sorana D. Bolboacă, Ioana C. Stănescu, Carmen A. Sfrângeu, Angelo C. Bulboacă

**Affiliations:** ^1^Department of Pathophysiology, Iuliu Haţieganu University of Medicine and Pharmacy Cluj-Napoca, Victor Babeş Str., No. 4-6, 400012 Cluj-Napoca, Romania; ^2^Department of Medical Informatics and Biostatistics, Iuliu Haţieganu University of Medicine and Pharmacy Cluj-Napoca, Louis Pasteur Str., No. 6, 400349 Cluj-Napoca, Romania; ^3^Department of Neurology and Pediatric Neurology, Iuliu Haţieganu University of Medicine and Pharmacy Cluj-Napoca, Victor Babeş Str., No. 43, 400012 Cluj-Napoca, Romania

## Abstract

**Objective:**

Our study aimed to investigate the analgesic and antioxidative stress effects of Curcumin (CC) in experimental migraine induced by Nitroglycerin (NTG) on rats, compared with Indomethacin (ID) and Propranolol (PP) treatments.

**Material and Methods:**

Five groups of 10 rats treated i.p. were investigated: control group (healthy rats) injected with saline solution (0.9%), NTG-control group injected with NTG (1 mg/100 gbw, bw = body weight), and three groups with pretreatment applied 30 min previous to the formalin test (NTG + CC group: Curcumin (10 mg/100 gbw), NTG + PP group: Propranolol (100 *μ*g/100 gbw), and NTG + ID group: Indomethacin (0.5 mg/100 gbw)). Formalin test was performed and number of flinches and shakes were counted. Several oxidative stress parameters were also assessed.

**Results:**

The smallest values of malondialdehyde (MDA), nitric oxide (NOx), and total oxidative status (TOS) were observed on NTG + CC with significant differences as compared with the control group (*p* < 0.0001). The group pretreated with Curcumin proved significantly smaller number of flinches and shakes compared with both NTG + PP and NTG + ID.

**Conclusion:**

Our study demonstrates a superior activity of Curcumin not only versus control, but also versus Propranolol and Indomethacin.

## 1. Introduction

Migraine is a common neurovascular disorder with uncomplete understood pathogenetic mechanisms. Pain modulation is an important issue in migraine research field. Therefore, understanding the pathogenesis of migraine and developing new treatments based on pathophysiological mechanisms are major issues in neuropharmacology. Several theories on migraine pathogenesis have been postulated in recent years, and vascular theory and migraine pain hypersensitivity are in the topic of migraine research field [1]. Migraine is associated with vasodilatation and blood-brain barrier permeabilization due to neurogenic inflammation [2]. One of the pathophysiological mechanisms in migraine with aura is known to be an excessive production of nitric oxide (which is a potent vasodilator molecule) even though the oxidative pathway changes in migraine are still uncertain [3]. Systemic administration of Nitroglycerin (NTG), a powerful vasodilator compound, is the most used experimental model of migraine [4]. NTG is a nitric oxide (NO) donor and has been proved to provoke spontaneous migraine-like attacks in migraine patients probably through sensitization phenomena and by facilitation of temporal summation of pain [5].

The theory of pain sensitization (including peripheral and central sensitization) has been postulated in migraine pathogenesis [6]. Peripheral sensitization is characterized by pulsatile headache exacerbation when migraine patients cough or perform activities, which may increase intracranial pressure, while the central sensitization is reflected by skin paralgesia in various regions of cephalic extremity and neck due to action of nonnoxious stimuli [7]. There are some structures which have been identified on animal experimental models and can contribute to migraine associated hyperalgesia. NTG produces hyperalgesia in rats through the activation of spinal and brainstem structures which contribute to nociception [8]. The hyperalgesia induced by systemic NTG administration is a well-established experimental model for somatic pain in rats [9]. Behavioral changes at formalin test after systemic NTG administration were used as an experimental model for various therapeutic substances (such as anandamide) [10].

Traditionally, the migraine treatment consists of treatment of the migraine attack and prophylaxis treatment. Acute treatment targets the serotoninergic system (such as triptans), the inflammatory reaction (nonsteroidal anti-inflammatory drugs), and calcitonin gene related protein (CGRP) receptors [11]. Some preventive drugs belong to the family of antiepileptic drugs (such as Topiramate and Valproate) [12, 13] or beta-1 blockers drugs (such as Propranolol and Metoprolol) [14]. A hyperexcitable brain state based on cortical spreading depression (CSD) has been postulated as a pathophysiological mechanism in migraine [15]. Therefore, normalizing neuronal firing and changing the threshold for neuronal discharge, based on modulation of CSD, are an important therapeutic target in migraine [16]. It has been shown that Valproate, Topiramate, and Propranolol inhibit the CSD in rats. Propranolol administration between migraine attacks is a common treatment for migraine patients that contributes to increased temporal distances between migraine attacks [17].

Curcumin (diferuloylmethane), a component of turmeric* (Curcuma longa),* is an inexpensive, orally bioavailable, and highly safe treatment in humans. Based on anti-inflammatory properties of Curcumin and on its potency to reduce the nitric oxide synthesis, our hypothesis was that this compound may have effects on the prophylaxis of migraine [18]. Propranolol is able to negatively modulate trigeminal nociception through *β*_1_ receptors antagonism on thalamocortical neurons. The VPM (ventral posteromedial) nucleus is a sensory processing centre for trigeminovascular nociception that is likely to be involved in migraine [19]. Indomethacin has been demonstrated to have an antimigraine effect due to its dual properties, antinociceptive, and anti-inflammatory potency [20]. Based on the above facts, we aimed to assess the protective effect on nociception of Curcumin on a migraine animal experimental model and to compare its effects with the effects of Propranolol (used in migraine attack prophylaxis) and Indomethacin (used for migraine attack treatment).

## 2. Material and Methods

### 2.1. Ethics Statement

All animal experiments were conducted in accordance with the protocols approved by the Ethics Committee of the Iuliu Haţieganu University of Medicine and Pharmacy Cluj-Napoca (291/26.05.2015).

### 2.2. Experimental Model

Wistar Bratislava albino male rats (from the Animal Department of Faculty of Medicine, Iuliu Haţieganu University of Medicine and Pharmacy Cluj-Napoca) were kept in polypropylene cages at constant temperature (24 ± 2°C), humidity (60 ± 5%), and light-dark regime, at the Department of Pathophysiology. The animals weighted 200–250 g and had free access to standard pellets as basal diet (Cantacuzino Institute, Bucharest, Romania) and water ad libitum.

The rats were randomly divided into 5 groups as follows, with 10 animals/group:

- Group 1 (control group): injected with saline solution (0.9%), 1 ml i.p
- Group 2 (NTG-control group): injected with NTG (1 mg/100 g body weight), 1 ml i.p. [21]
- Group 3 (NTG + CC): injected with NTG (1 mg/100 g body weight), 1 ml i.p. + pretreatment with Curcumin (10 mg/100 g body weight) [22]
- Group 4 (NTG + PP): injected with NTG (1 mg/100 g body weight), 1 ml i.p + pretreatment with Propranolol (100 *μ*g/100 g body weight) [23]
- Group 5 (NTG + ID): injected with NTG (1 mg/100 g body weight), 1 ml i.p + Indomethacin injected i.p., 0.5 mg/100 g body weight i.p., 30 min previous to the formalin test [24]

The pretreatment with Curcumin or Propranolol was done daily by gavage, 14 days previous to NTG administration. Curcumin was dissolved in groundnut oil as described by Joe et al. [25]. Propranolol was dissolved in 1 ml saline 0.9% before administration. Nitroglycerin, Propranolol, Curcumin, and Indomethacin were procured from Sigma-Aldrich (Bio-Zyme, Romania).

Formalin test is a reliable and valid model of nociception and was applied as previously described [9]. The noxious stimulus was an injection of diluted formalin (1% in saline) under the skin of the right hind paw; after that, the animals were placed in plexiglass cages for observation. The total number of flinches (rapid and brief withdraw or flexion of the injected paw) per min was counted during two distinct phases. The first phase consisted in immediate assessment after formalin administration, during the period of 1–5 min after injection. The second phase consisted in assessment for 1 min periods at 5-min intervals during 10–60 min after formalin administration. The data were collected as total flinches and shakes per each phase of formalin test. The formalin test permits the assessment of rat's behavior during these two distinct phases. The animals were kept under the observation during the period between NTG, saline administration, and formalin test (4 h). Phase I is dominated by vasodilatation effects of noxious stimulus, while phase II is dominated by the inflammatory mechanisms induced by the noxious stimulus [26].

### 2.3. Measurements

#### 2.3.1. Blood Pressure

The systolic arterial pressure (SBP) in conscious, nonanesthetized rats was measured by tail-cuff plethysmography (BIOPAC system 3.7.7). The pressure was monitored before (SBP baseline) NTG/saline administration (30 min previous the experiment) and 30 min before formalin administration (SBP follow-up). Three measurements were made for each moment of experiment. The average was calculated and recorded.

#### 2.3.2. Blood Samples and Serum Analysis

Blood samples were collected from the tail vein previous experiment. At the end of experiment, the blood samples were collected from the retroorbital plexus of each rat under light ketamine anesthesia (5 mg/100 g body weight i.p. route) [27]. The animals were euthanatized by cervical dislocation after this procedure.

The oxidative stress parameters measurements were made as follows: malondialdehyde (MDA) according to Yagl method [28] and thiol compound according to Ellman method [29]. The indirect assessment of NO synthesis (NOx), total oxidative capacity (TOS), and total oxidative status (TAC) were made following the method described by Parvu et al. [30]. The MDA, NOx, and TOS represented the evaluation of oxidative stress intensity, and the assessment of thiol and TAC represented the evaluation of antioxidant capacity of the blood. All the spectroscopic measurements were performed using Jasco V-350 UV-VIS spectrophotometer (Jasco International Co., Ltd., Tokyo, Japan).

### 2.4. Statistical Analysis

Measured data were expressed as the mean ± standard deviation. Comparisons between two of groups (such as each group with control group and groups with different treatments as PP, CC, and ID) were done with Student's* t*-test for independent samples. Two-tailed tests were used for all comparisons and the *p* values less than 0.05 were considered statistically significant. The statistical analysis was done with Statistica 8 (StatSoft, USA).

## 3. Results

The smallest values of MDA, NOx, and TOS were observed on NTG + CC (Table 1) with significant differences as compared with the control group (*p* < 0.0001). With few exceptions observed for TAC, significant differences were observed when the values of anti- and prooxidants of each group were compared with the control group, as well as when the values of groups with pretreatments (NTG + PP, NTG + CC, and NTG + ID) were compared with NTG group (*p* < 0.002). No significant differences were observed when the values of TAC in NTG + ID group were compared with those in control group (*p* = 0.4069) or NTG group (*p* = 0.6790). All other comparisons between groups proved statistically significant (*p* < 0.05).

As expected, the smallest values of systolic blood pressure in both evaluations were observed on NTG + PP group. Significant differences between groups as presented in Table 2 were observed (Table 2).

Number of flinches and shakes in the first phase varied between 19 and 45, with the smallest values observed on control group (from 19 to 22), closely followed by the NTG + CC (from 25 to 28), and the highest values on NTG group (from 39 to 45). The same trend was observed also on phase two, with the smallest values observed on control group (from 109 to 115), closely followed by the NTG + CC (from 120 to 124), and the highest values on NTG group (from 150 to 155). Descriptive values of flinches and shakes by groups are presented in Table 3.

**Figure 1:**
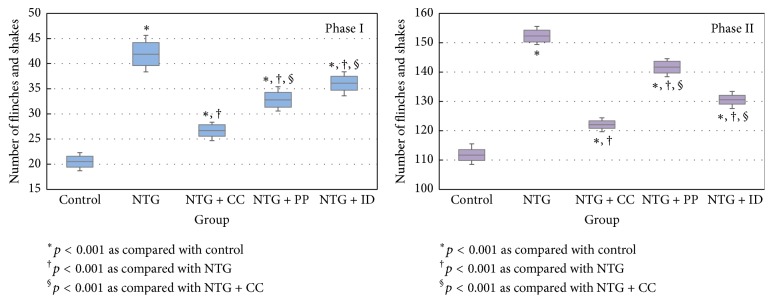
Number of flinches and shakes expressed as mean (the high of the column) and standard deviation (bars above the column) and different comparisons between groups for the two investigated phases.

The smallest values of number of flinches and shakes were observed on the control group and the differences between these groups were statistically significant (*p* < 0.001; see Figure 1). Furthermore, the NTG group proved significantly higher number of flinches and shakes as compared with all other groups (NTG + CC, NTG + PP, and NTG + ID). The group pretreated with Curcumin proved significantly smaller number of flinches and shakes compared with both NTG + PP and NTG + ID (Figure 1).

## 4. Discussions

The nociceptive effect of formalin injected in the rat hind paw consists in two phases, with each being characterized by different pathophysiological mechanisms. The nociception in the first phase is due to direct action of noxious stimulus on nociceptors, while in the second phase, the nociceptive process is dominated by inflammatory mechanism [26]. Phase I has the associated gate control mechanism (C-fiber inhibition), and in phase II the sensitization process is dominated by the inflammatory mediators [9].

Centrally acting analgesics were proved to inhibit both phases of formalin test [31], while nonsteroidal anti-inflammatory drugs can inhibit only the late phase (e.g., Indomethacin) [32] or both phases (e.g., acetylsalicylic acid) [33].

Several studies showed that the Nitroglycerin induces hyperalgesia in rats in the same manner but on different animal species and weight (Sprague-Dawley rats, weight 180–200 mg) [34, 35]. Ruggieri et al. introduced an experimental model of hot-plate induced nociception and on formalin test induced hyperalgesia on Wistar rats that can be used for testing the efficiency of different compounds on nociception pathophysiological mechanisms [36]. One of the possible mechanisms associated with hyperalgesia after NTG administration consists in central sensitization induced by NO released from NTG. NO produced the increase of amino-acids neurotransmitters such as glutamate, which is a signaling molecule for the central sensitization [37]. NO derived from NTG is believed to mimic the same biological effects like endogenous NO [38].

### 4.1. Propranolol Effect

Propranolol is widely used as a nonselective *β*-receptor antagonist with proved efficiency in migraine prevention [39]. Chronic administration of Propranolol significantly reduces the cortical spreading depression (CSD) involved in migraine attacks [40]. Propranolol has the potency to reduce the neuronal firing due to its membrane-stabilizing properties and modulation of L-glutamate action [19]. Pathophysiological mechanisms of Propranolol can be partly due to modulation of vasodilatation associated with inflammatory process. According to some authors, hemodynamic changes, with blood flow reduction, associated with adrenoreceptor agonist administration exist [41, 42]. In addition to its action on *β*-adrenoreceptors, Propranolol may act as antagonist of 5-HT1A/1B/1D receptors [43]. The reduction of oxidative stress by beta-blockers can contribute to the antinociceptive effect. Gomes et al. demonstrated that the beta-blockers, including Propranolol, have scavenging activity for reactive oxygen species (ROS): (O(2)(−), H(2)O(2), HO(*∙*), HOCl, and ROO(*∙*) and for reactive nitrogen species (RNS): (*∙*)NO and ONOO(−) [44]. In our study, Propranolol pretreatment induced a decrease in MDA and TOS (Table 1) and decrease in blood pressure (Table 2) and had an antinociceptive effect in both phases (Table 3, Figure 1). The Propranolol effect was more intense in phase I by acting on beta-adrenergic receptors on the vessels wall. In phase II, as the increase of vessels permeability due to inflammation occurred, the Propranolol effect was reduced. Even though the TAC was not influenced, the oxidative stress parameters were reduced by Propranolol administration (Table 1).

### 4.2. Indomethacin Effect

Migraine associated hyperalgesia is partially mediated by the increase of prostaglandin production, NO production, and vasoactive peptides that can act on afferent nociceptors and nociception pathways. The synthesis of prostanoids and vasoactive peptides follows early activation of the L-arginine/NO pathway. Prostanoids have algogenic and vasoactive properties that contribute to pain pathophysiology [45]. Some mechanisms have been proposed for the antinociceptive effect of Indomethacin. Indomethacin can reduce the effect of substance P and N-methyl-D-aspartate on the spinal dorsal horn neurons. Pitcher and Henry were first who linked the prostanoids and possibly arachidonic acid and other eicosanoids to the effects of substance P and glutamate in the spinal dorsal horn [46]. Immediately after formalin injection, there is an activation of constitutive spinal COX-2 [47] and the substance P production is enhanced [48]. Our results demonstrated that Indomethacin treatment influences the nociception in phase II when the inflammation occurred but had no significant effect on phase I (Table 3, Figure 1). It also reduced the oxidative stress parameters but had no significant effect on TAC and Thiol (Table 1).

### 4.3. Curcumin Effect

Curcumin has been intensively studied for its multiple biological actions including anti-inflammatory and antioxidant activities. Mechanisms contributing to these effects are under research. Previous reports have demonstrated the analgesic effects of systemic administration of Curcumin in various experimental models such as formalin-induced orofacial pain [49], capsaicin-induced thermal hyperalgesia, or mechanical and thermal hyperalgesia induced by chronic constrictive injury [50]. Other authors demonstrated its analgesic effect by intrathecal administration [51, 52]. The results of our study demonstrated that the Curcumin has an analgesic effect in both phases of formalin test (Table 3, Figure 1). Therefore, it can be considered that the Curcumin has the ability to modulate the activity in the primary afferent fibers induced by formalin in phase I and to reduce the activation of wide dynamic range neurons in dorsal horn. The hyperactivity of wide dynamic range neurons which represents a facilitated pain state, despite reduction of pain stimuli, is a feature of phase II of formalin test [53]. It is suggested that the early phase is due to a direct effect on nociceptors and the late phase seems to be an inflammatory response with inflammatory pain [26].

Systemic administration of Curcumin was more beneficial than Indomethacin or Propranolol administration regarding the decrease of the oxidative stress parameters and nociceptive process. The oxidative stress parameters measurements were significantly improved after Curcumin treatment compared with Indomethacin and Propranolol treatment groups. Comparative effect of Curcumin with other drugs was assessed by Di Pierro et al. who demonstrated the superiority of Curcumin compared with Acetaminophen in clinical study for acute pain of various origins proving that the analgesic properties of Curcumin have a clinical translation [54]. Our hypothesis in this study was that the antinociceptive effect of Curcumin is mediated in part by its antioxidative properties. The smallest values of MDA, NOx, and TOS were observed on all experimental groups with Curcumin administration (Table 1). Curcumin also reduced the nociceptive response in both phases I and II. Ciftci et al. demonstrated that Curcumin could reduce the level of MDA and significantly increase the TAC, reducing the oxidative DNA damage in cells [55]. The level of oxidative stress is partially reduced due to its scavenging potential. Oxidative stress mediators such as hydroxyl radical (*∙*OH) can be scavenged by Curcumin [56]. The antinociceptive activity of Curcumin is possible through its inhibitory action on nitro-oxidative stress, reducing NO synthesis and through its anti-inflammatory properties such as the reduction of TNF-alpha release [57]. The decrease in blood pressure due to Curcumin administration was also observed in our experimental study (Table 2), in the context of existing evidence that oxidative stress can produce neurogenic hypertension by influencing the central nervous system [58]. The numbers of flinches and shakes reported in our study (Table 3) are significantly lower (ranging from 3.80% to 5.44% of the mean values for the first phase and from 1.06% to 1.64% of the mean values for the second phase) than those in comparable studies [34–36] with experiments conducted on different breeds of rats. The differences among investigated groups sustain the superiority of the Curcumin activity not only versus control group, but also versus Propranolol and Indomethacin groups. Therefore, these results added to other data existing in literature can be hopeful for adding the Curcumin as prophylaxis in migraine, to other drugs already in use, for its beneficial effect. Curcumin needs further studies for elucidation of the mechanisms of pain decrease in experimental migraine.

## 5. Conclusions

Preemptive analgesic effect was demonstrated for Curcumin, administrated before pain stimuli. Pretreatment with Curcumin decreased the nociception in rats, after Nitroglycerin administration in both phases, phase I dominated by vasodilatation and phase II dominated by inflammatory reaction. The decrease in oxidative stress parameters and blood pressure was also obtained after Curcumin administration. The differences among investigated groups sustain the superiority of the Curcumin activity not only versus control group, but also versus Propranolol and Indomethacin groups.

## Figures and Tables

**Table 1:** Mean ± standard deviation for stress oxidative parameters by groups.

Group	MDA	NOx	TOS	Thiol	TAC
Control	2.57 ± 0.07	14.80 ± 2.39	26.30 ± 1.16	205.30 ± 2.95	1.23 ± 0.04
NTG	4.52 ± 0.08	35.50 ± 1.27	45.10 ± 1.52	182.30 ± 3.23	1.11 ± 0.03
NTG + PP	3.24 ± 0.14	37.90 ± 1.66	36.70 ± 1.49	193.80 ± 3.29	1.19 ± 0.02
NTG + CC	2.33 ± 0.03	22.40 ± 1.26	31.70 ± 1.89	177.40 ± 4.20	1.60 ± 0.03
NTG + ID	4.20 ± 0.02	32.90 ± 1.29	35.90 ± 1.66	194.50 ± 5.48	1.15 ± 0.29

MDA = malondialdehyde; NOx = nitric oxide; TOS = total oxidative status; thiol = thiol compound; TAC = total antioxidative capacity.

**Table 2:** Systolic blood pressure expressed as mean ± standard deviation before and after saline solution/NTG administration.

Group	TAS baseline	TAS follow-up
Control	117.00 ± 2.11	127.30 ± 4.11
NTG	116.70 ± 1.57	95.00 ± 3.16^h^
NTG + PP	112.40 ± 1.90^a,d,g^	88.30 ± 3.02^h,i,j^
NTG + CC	120.00 ± 3.09^b,e^	110.20 ± 4.13^h,i^
NTG + ID	120.60 ± 2.32^c,f^	106.90 ± 3.57^h,i^

Compared with control group: ^a^*p* = 0.001, ^b^*p* = 0.0207, ^c^*p* = 0.0019, and ^h^*p* < 0.0002; compared with NTG group: ^d^*p* < 0.001, ^e^*p* = 0.0075, ^f^*p* = 0.0003, and ^i^*p* < 0.0002; compared with NTG + CC group: ^g^*p* < 0.001, ^j^*p* < 0.001.

**Table 3:** Statistic summary of formalin test.

Group	Phase I			Phase II		
	Mean ± SD	SE	Median [*Q*1–*Q*3]	Mean ± SD	SE	Median [*Q*1–*Q*3]
Control	20.50 ± 1.08	0.34	20.50 [20.00–21.00]	111.70 ± 1.83	0.58	111.50 [110.25–113.00]
NTG	41.90 ± 2.28	0.72	41.50 [40.25–43.75]	152.30 ± 2.00	0.63	152.50 [150.25–153.75]
NTG + PP	26.70 ± 1.16	0.37	27.00 [26.00–27.75]	122.10 ± 1.29	0.41	122.00 [121.25–122.75]
NTG + CC	32.80 ± 1.48	0.47	33.00 [31.25–34.00]	141.70 ± 2.00	0.63	141.50 [140.25–143.75]
NTG + ID	36.10 ± 1.37	0.43	36.00 [35.00–37.00]	130.60 ± 1.51	0.48	130.50 [130.00–131.75]

NTG = Nitroglycerin; PP = Propranolol; CC = Curcumin; ID = Indomethacin; SD = standard deviation; SE = standard error of the mean; *Q*1 = first quartile (25th percentile); *Q*3 = third quartile (75th percentile).

## References

[1] Gasparini C. F., Sutherland H. G., Griffiths L. R. (2013). Studies on the pathophysiology and genetic basis of migraine. *Current Genomics*.

[2] DosSantos M. F., Holanda-Afonso R. C., Lima R. L., DaSilva A. F., Moura-Neto V. (2014). The role of the blood–brain barrier in the development and treatment of migraine and other pain disorders. *Frontiers in Cellular Neuroscience*.

[3] Neri M., Frustaci A., Milic M. (2015). A meta-analysis of biomarkers related to oxidative stress and nitric oxide pathway in migraine. *Cephalalgia*.

[4] Buzzi M. G., Tassorelli C. (2010). Experimental models of migraine. *Handbook of Clinical Neurology*.

[5] De Tommaso M., Libro G., Guido M. (2004). Nitroglycerin induces migraine headache and central sensitization phenomena in patients with migraine without aura: A study of laser evoked potentials. *Neuroscience Letters*.

[6] Biondi D. M. (2006). Is migraine a neuropathic pain syndrome?. *Current Pain and Headache Reports*.

[7] Zhang Y., Shao G., Zhang W. (2013). Gabapentin inhibits central sensitization during migraine. *Neural Regeneration Research*.

[8] Buzzi M. G., Tassorelli C., Nappi G. (2003). Peripheral and central activation of trigeminal pain pathways in migraine: Data from experimental animal models. *Cephalalgia, Supplement*.

[9] Tjølsen A., Berge O.-G., Hunskaar S., Rosland J. H., Hole K. (1992). The formalin test: an evaluation of the method. *PAIN*.

[10] Greco R., Mangione A. S., Sandrini G., Maccarrone M., Nappi G., Tassorelli C. (2011). Effects of anandamide in migraine: Data from an animal model. *The Journal of Headache and Pain*.

[11] Gilmore B., Michael M. (2011). Treatment of acute migraine headache. *American Family Physician*.

[12] Salisbury-Afshar E. (2014). Topiramate for the prophylaxis of episodic migraine in adults. *American Family Physician*.

[13] Factor S. A., Jankovic J., Friedman B. W., Garber L., Gallagher E. J. (2014). Randomized trial of iv valproate vs metoclopramide vs ketorolac for acute migraine. *Neurology*.

[14] Evers S., Áfra J., Frese A. (2009). EFNS guideline on the drug treatment of migraine—revised report of an EFNS task force. *European Journal of Neurology*.

[15] Casucci G., Villani V., Frediani F. (2008). Central mechanism of action of antimigraine prophylactic drugs. *Neurological Sciences*.

[16] Dussor G. (2015). ASICs as therapeutic targets for migraine. *Neuropharmacology*.

[17] Freitag F. G. (2007). The cycle of migraine: patients' quality of life during and between migraine attacks. *Clinical Therapeutics*.

[18] Bulboacă A., Bolboacă S. D., Suciu H. S. (2016). Protective effect of curcumin in fructose-induced metabolic syndrome and in streptozotocin-induced diabetes in rats. *Iranian Journal of Basic Medical Sciences*.

[19] Shields K. G., Goadsby P. J. (2005). Propranolol modulates trigeminovascular responses in thalamic ventroposteromedial nucleus: A role in migraine?. *Brain*.

[20] Saxena P. R., Den Boer M. O. (1991). Pharmacology of antimigraine drugs. *Journal of Neurology*.

[21] Tassorelli C., Greco R., Wang D., Sandrini G., Nappi G. (2006). Prostaglandins, glutamate and nitric oxide synthase mediate nitroglycerin-induced hyperalgesia in the formalin test. *European Journal of Pharmacology*.

[22] Nonose N., Pereira J. A., Machado P. R. M., Rodrigues M. R., Sato D. T., Martinez C. A. R. (2014). Oral administration of curcumin (Curcuma longa) can attenuate the neutrophil inflammatory response in zymosan-induced arthritis in rats. *Acta Cirurgica Brasileira*.

[23] Valdivia L. F., Centurión D., Perusquía M., Arulmani U., Saxena P. R., Villalón C. M. (2004). Pharmacological analysis of the mechanisms involved in the tachycardic and vasopressor responses to the antimigraine agent, isometheptene, in pithed rats. *Life Sciences*.

[24] Owoyele B. V., Oladejo R. O., Ajomale K., Ahmed R. O., Mustapha A. (2014). Analgesic and anti-inflammatory effects of honey: The involvement of autonomic receptors. *Metabolic Brain Disease*.

[25] Joe B., Nagaraju A., Gowda L. R., Basrur V., Lokesh B. R. (2014). Mass-spectrometric identification of T-kininogen I/thiostatin as an acute-phase inflammatory protein suppressed by curcumin and capsaicin. *PLoS ONE*.

[26] Hunskaar S., Hole K. (1987). The formalin test in mice: dissociation between inflammatory and non-inflammatory pain. *PAIN*.

[27] Amani M., Ali N., Reza B., Ali K. (2010). Effect of ascorbic acid supplementation on nitric oxide metabolites and systolic blood pressure in rats exposed to lead. *Indian Journal of Pharmacology*.

[28] Aebi H. (1984). Assay for blood plasma and serum peroxides. *Methods in Enzymology*.

[29] Ellman G. L. (1958). A colorimetric method for determining low concentrations of mercaptans. *Archives of Biochemistry and Biophysics*.

[30] Parvu A. E., Parvu M., Vlase L., Miclea P., Mot A. C., Silaghi-Dumitrescu R. (2014). Anti-inflammatory effects of Allium schoenoprasum L. leaves. *Journal of Physiology and Pharmacology*.

[31] Segawa T., Miyakoshi N., Kasukawa Y., Aonuma H., Tsuchie H., Shimada Y. (2013). Analgesic effects of minodronate on formalin-induced acute inflammatory pain in rats. *Biomedical Research (Japan)*.

[32] Ortiz M. I. (2012). Metformin and phenformin block the peripheral antinociception induced by diclofenac and indomethacin on the formalin test. *Life Sciences*.

[33] Pini L.-A., Vitale G., Sandrini M. (1997). Serotonin and opiate involvement in the antinociceptive effect of acetylsalicylic acid. *Pharmacology*.

[34] Tassorelli C., Greco R., Wang D., Sandrini M., Sandrini G., Nappi G. (2003). Nitroglycerin induces hyperalgesia in rats - A time-course study. *European Journal of Pharmacology*.

[35] Greco R., Siani F., Demartini C. (2016). Andrographis paniculata shows anti-nociceptive effects in an animal model of sensory hypersensitivity associated with migraine. *Functional Neurology*.

[36] Ruggieri V., Vitale G., Filaferro M., Frigeri C., Pini L. A., Sandrini M. (2010). The antinociceptive effect of acetylsalicylic acid is differently affected by a CB1 agonist or antagonist and involves the serotonergic system in rats. *Life Sciences*.

[37] Merino J. J., Arce C., Naddaf A., Bellver-Landete V., Oset-Gasque M. J., González M. P. (2014). The nitric oxide donor SNAP-induced amino acid neurotransmitter release in cortical neurons. Effects of blockers of voltage-dependent sodium and calcium channels. *PLoS ONE*.

[38] Ma S., Long J. P. (1991). Central noradrenergic activity is responsible for nitroglycerin-induced cardiovascular effects in the nucleus tractus solitarii. *Brain Research*.

[39] Shimizu T. (2009). *β* blockers in migraine prophylaxis. *Brain and Nerve*.

[40] Mathew N. T. (2011). Pathophysiology of chronic migraine and mode of action of preventive medications. *Headache: The Journal of Head and Face Pain*.

[41] Gardiner S. M., March J. E., Kemp P. A., Bennett T. (2005). Involvement of CB1-receptors and *β*-adrenoceptors in the regional hemodynamic responses to lipopolysaccharide infusion in conscious rats. *American Journal of Physiology-Heart and Circulatory Physiology*.

[42] Koeda T., Sato J., Kumazawa T., Tsujii Y., Mizumura K. (2002). Effects of adrenoceptor antagonists on the cutaneous blood flow increase response to sympathetic nerve stimulation in rats with persistent inflammation. *The Japanese Journal of Physiology*.

[43] Nishio H., Nagakura Y., Segawa T. (1989). Interactions of carteolol and other *β*-adrenoceptor blocking agents with serotonin receptor subtypes. *Archives Internationales de Pharmacodynamie et de Thérapie*.

[44] Gomes A., Costa D., Lima J. L., Fernandes E. (2006). Antioxidant activity of beta-blockers: an effect mediated by scavenging reactive oxygen and nitrogen species?. *Bioorganic Medicinal Chemistry*.

[45] Sarchielli P., Alberti A., Codini M., Floridi A., Gallai V. (2000). Nitric oxide metabolites, prostaglandins and trigeminal vasoactive peptides in internal jugular vein blood during spontaneous migraine attacks. *Cephalalgia*.

[46] Pitcher G. M., Henry J. L. (1999). Mediation and modulation by eicosanoids of responses of spinal dorsal horn neurons to glutamate and substance P receptor agonists: Results with indomethacin in the rat in vivo. *Neuroscience*.

[47] Yamamoto T., Nozaki-Taguchi N. (1996). Analysis of the effects of cyclooxygenase (COX)-1 and COX-2 in spinal nociceptive transmission using indomethacin, a non-selective COX inhibitor, and NS-398, a COX-2 selective inhibitor. *Brain Research*.

[48] Herzberg U., Hama A., Sagen J. (2008). Spinal subarachnoid adrenal medullary transplants reduce hind paw swelling and peripheral nerve transport following formalin injection in rats. *Brain Research*.

[49] Mittal N., Joshi R., Hota D., Chakrabarti A. (2009). Evaluation of antihyperalgesic effect of curcumin on formalin-induced orofacial pain in rat. *Phytotherapy Research*.

[50] Di Y. X., Hong C., Jun L., Renshan G., Qinquan L. (2014). Curcumin attenuates mechanical and thermal hyperalgesia in chronic constrictive injury model of neuropathic pain. *Pain and Therapy*.

[51] Lee J. H., Kim Y. D., Jung H. C., Cheong Y. K. (2014). The effect of intrathecal curcumin on mechanical allodynia in rats after L5 spinal nerve ligation. *Korean Journal of Anesthesiology*.

[52] Han Y. K., Lee S. H., Jeong H. J., Kim M. S., Yoon M. H., Kim W. M. (2012). Analgesic effects of intrathecal curcumin in the rat formalin test. *The Korean Journal of Pain*.

[53] Puig S., Sorkin L. S. (1996). Formalin-evoked activity in identified primary afferent fibers: systemic lidocaine suppresses phase-2 activity. *PAIN*.

[54] Di Pierro F., Rapacioli G., Di Maio E. A., Appendino G., Franceschi F., Togni S. (2013). Comparative evaluation of the pain-relieving properties of a lecithinized formulation of curcumin (Meriva®), nimesulide, and acetaminophen. *Journal of Pain Research*.

[55] Ciftci G., Aksoy A., cenesiz S. (2015). Therapeutic role of curcumin in oxidative DNA damage caused by formaldehyde. *Microscopy Research and Technique*.

[56] Borra S. K., Mahendra J., Gurumurthy P., Jayamathi, Iqbal S. S., Mahendra L. (2014). Effect of curcumin against oxidation of biomolecules by hydroxyl radicals. *Journal of Clinical and Diagnostic Research*.

[57] Sharma S., Kulkarni S. K., Agrewala J. N., Chopra K. (2006). Curcumin attenuates thermal hyperalgesia in a diabetic mouse model of neuropathic pain. *European Journal of Pharmacology*.

[58] Huang Y.-P., Jin H.-Y., Yu H.-P. (2017). Inhibitory effects of alpha-lipoic acid on oxidative stress in the rostral ventrolateral medulla in rats with salt-induced hypertension. *International Journal of Molecular Medicine*.

